# Charcot-Marie-Tooth neuropathy type 2A: novel mutations in the mitofusin 2 gene (*MFN2*)

**DOI:** 10.1186/1471-2350-7-53

**Published:** 2006-06-08

**Authors:** Kathrin Engelfried, Matthias Vorgerd, Michaela Hagedorn, Gerhard Haas, Jürgen Gilles, Jörg T Epplen, Moritz Meins

**Affiliations:** 1 Department of Human Genetics, Ruhr-University Bochum, Germany; 2 Department of Neurology, Neuromuscular Center Ruhrgebiet, Ruhr-University Bochum, Germany; 3 Neurology, Evangelische Stiftung Tannenhof, Remscheid, Germany; 4 Neurology, St.-Marien-Hospital, Lünen, Germany

## Abstract

**Background:**

Charcot-Marie-Tooth neuropathies are a group of genetically heterogeneous diseases of the peripheral nervous system. Mutations in the *MFN2 *gene have been reported as the primary cause of Charcot-Marie-Tooth disease type 2A.

**Methods:**

Patients with the clinical diagnosis of Charcot-Marie-Tooth type 2 were screened using single strand conformation polymorphism (SSCP). All DNA samples showing band shifts in the SSCP analysis were amplified from genomic DNA and cycle sequenced.

**Results:**

We analyzed a total of 73 unrelated patients with a clinical diagnosis of CMT 2. Overall, novel mutations were detected in 6 patients. c.380G>T (G127V), c.1128G>A (M376I), c.1040A>T (E347V), c.1403G>A (R468H), c.2113G>A (V705I), and c.2258_2259insT (L753fs).

**Conclusion:**

We confirmed a significant role of mutations in *MFN2 *in the pathogenesis of Charcot-Marie-Tooth disease type 2.

## Background

Charcot-Marie-Tooth neuropathies (CMT), also named as hereditary motor and sensory neuropathies (HMSN) are a group of genetically heterogeneous diseases of the peripheral nervous system. CMT has been classified into two major subgroups. A severe reduction of nerve conduction velocities (NCV) with NCV < 38 m/s is found in the demyelinating CMT type 1. The axonal type (CMT type 2) is characterized by preferential degeneration of the axon showing amplitude reductions in nerve conduction studies but only mildly reduced NCV. Both CMT1 and CMT2 have been recognized to be genetically heterogeneous. Several genetic loci have been defined for both clinical types. CMT2A was located to chromosome 1p35–p36 [1-3]. Although a mutation in *KIF1B *was published in a large CMT2A family, no further mutations were detected in other families with CMT2A [4,5]. Züchner *et al*. identified the *MFN2 *gene by means of linkage studies and reported mutations in *MFN2 *in seven large families with linkage to the CMT2A locus [5]. Recently, other groups confirmed the *MFN2 *gene as the primary cause of CMT2A [6-8]. We screened 73 unrelated patients with the clinical diagnosis of CMT2 and identified six new disease-causing mutations.

## Methods

### Subjects

We analyzed samples of 73 unrelated patients sent to our diagnostic laboratory with the request of genetic analysis for Charcot-Marie-Tooth disease. Linkage analysis was not possible in these individuals. Inclusion criteria for the *MFN2 *analysis were a clinical classification of CMT2, nerve conduction velocity (NCV) > 38 m/s, or histological findings of axonal degeneration. EDTA blood samples were taken from patients after informed consent. DNA was extracted from blood leukocytes by standard methods. All control samples used to check the distribution of sequence alterations mentioned in the results sections were taken from an anonymous collective of ethnically matched samples.

### Mutation analysis of MFN2 gene

For mutation analysis in *MFN2*, primers were designed to amplify all exons including flanking intronic regions. Primer sequences are available on request. Exon 7 and 8, and exon 10 and 11, respectively, were each amplified in one amplicon including the intermediate intron. PCRs were performed in 96-well microtiter plates (Thermowell Costar Corning, NY) using a thermocycler (Biometra, Goettingen, Germany). Each well contained 50 ng DNA in 10 μl reaction volume, GC buffer (Genecraft, Münster, Germany), 10 pMol of forward and reverse primer, 1 U Taq Polymerase (Genecraft, Münster, Germany), 2 mMol of each dNTP, and a MgCl_2 _concentration of 1 mM. For SSCP analysis, 0.06 μl of [α^32 ^P] dCTP (10 mCi/ml) was included in the PCR. PCR conditions included initial denaturation (2 min at 94°C), two initial cycles at 94°C (15 s) and 6°C and 3°C above the main annealing temperature (30 s) followed by 30 s at 72°C, and 28–32 cycles of 94°C (15 s), annealing temperature (30 s) and final elongation step at 72°C (30 s). Annealing temperature was 62°C except for exons 7/8, 10/11, 15, 16, 17, and 8 (58°C). PCR products were digested with suitable restriction enzymes depending on the fragment size to optimize the mutation detection rate by SSCP analysis. In the SSCP analysis the denatured PCR products were separated by polyacrylamide (PAA) gel electrophoresis using two different conditions: 30% PAA (acrylamide/bisacrylamide: 19/1) gel, 1xTBE, and either 10% glycerol or 5% glycerol/1 M urea. Electrophoresis was carried out at 55 W for 3–4 h at 4°C. Gels were evaluated by autoradiography or exposure to a phosphoimager screen, using the corresponding software. Exon 13 was screened using SSCP and DHPLC. DNA samples showing band shifts in the SSCP analysis were amplified from genomic DNA and cycle sequenced by standard protocols using the Megabace 1000 (Amersham Bioscience, Freiburg, Germany). If possible, restriction analysis with a suitable restriction enzyme was applied to confirm the mutation and to analyse control samples. Enzymes were ordered from New England Biolabs if not indicated otherwise.

## Results

We analyzed a total of 73 unrelated patients with a clinical diagnosis of CMT 2. Overall, novel mutations were detected in 6 patients. The clinical features and the neurographical data of these patients are summarized (tables 1 and 2). Nerve biopsy has not been performed in any of these 6 patients.

**Table 1:** Clinical features of patients with *MFN2 *mutation

	**1**	**2**	**3**	**4**	**5**	**6**
**Age (y)/sex**	58/F	34/F	32/M	27/F	44/F	65/M
**Onset age (y)**	48	22	childhood	26	6	62
**Initial symptoms**	weakness in LE	muscle crampi in LE	-	paraesthesia in LE	foot deformities	gait ataxia
						
**Motor**						
**UE**	0	0	0	0	2+	0
**LE**	2+	2+	2+	2+	3+	2+
						
**Sensory**						
**pain/touch**	1+	1+	0	0	2+	2+
**deep sense**	2+	1+	-	1+	2+	2+
						
**Reflexes**	normal in UE, decreased (knee), absent (ankle)	normal in UE, absent (knee, ankle)	normal in UE, absent (ankle)	normal in UE, absent (knee, ankle)	decreased in UE, absent (knee, ankle)	normal in UE, absent (knee, ankle)
						
**Pes cavus/varus**	yes	yes	yes	yes	yes	yes

0, 1+, 2+, and 3+ = no, minimal, moderate, and severe involvement for muscle weakness and sensory deficit; – = no precise data. UE = upper extremities, LE = lower extremities.

**Table 2:** Nerve conduction study in patients with CMT associated with mutation in *MFN2*

	**Normal**	**1**	**2**	**3**	**4**	**5**	**6**
**Motor**							
*Median nerve*							
DML (ms)	≤ 3,9	2,8	3		3,8	5	
NCV (m/s)	≥ 50	53	50	n.d.	46,2	56	n.d.
Amplitude (mV)	≥ 6	11,4	14,2		n.d.	11	
*Peroneal nerve*							
DML (ms)	≤ 4,8	**7,6**	4		**5,3**		
NCV (m/s)	≥ 42	**37**	45	**not recordable**	47,8	**not recordable**	**not recordable**
Amplitude (mV)	≥ 4	**0,8**	8,4		**2**		
*Tibial nerve*							
DML (ms)	≤ 5,1	**6,2**	**4,2**	**7,1**			
NCV (m/s)	≥ 41	**31**	**36**	**38**	n.d.	**not recordable**	**not recordable**
Amplitude (mV)	≥ 5	**0,6**	**0,4**	**0,4**			

**Sensory**							
*Median nerve*							
NCV (m/s)	≥ 47	60	54	n.d.	57,7	**45**	n.d.
Amplitude (mV)	≥ 7	13,4	11		n.d.	20	
*Sural nerve*							
NCV (m/s)	≥ 41	**34**	**not recordable**	**30**	56,5	**32**	**not recordable**
Amplitude (mV)	≥ 10	**7,2**		**1**	n.d.	**6**	

DML= distal motor latency; NCV = nerve conduction velocity; n.d. = not determined.

**Figure 1:**
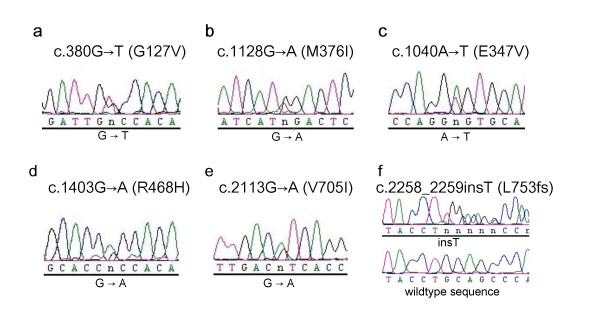
Electropherograms showing novel mutations in the *MFN2 *gene detected in this analysis. Numbering of nucleotides is according to the open reading frame of the cDNA sequence as deposited in GenBank (GenBank accession no. BC017061).

The heterozygous point mutation c.380 G>T in exon 5 of *MFN2 *(fig. 1a) was detected in a 58 year old female patient (patient 1), predicting the exchange of Glycine to Valine at position 127 of the MFN2 protein (G127V). The mutation leads to a loss of a recognition site for the restriction enzyme *Msc *I, which was used to exclude this mutation in 97 control persons (194 control chromosomes). Clinically the patient shows a predominant axonal neuropathy with normal motor nerve conduction velocity (mNCV) in electrophysiological studies of the median nerve. The family history is compatible with autosomal-dominant inheritance, but other family members were not available for genetic analysis.

Patient 2, a 34 year old male, is heterozygous for the point mutation c.1128G>A in exon 11, predicting the exchange of Methionine to Isoleucine at position 376 (M376I) (fig. 1b). This mutation is detectable by a loss of a *Nla*III restriction site, and was excluded by restriction analysis in 190 control chromosomes. The patient has distal sensory loss in his legs, absent Achilles' tendon reflexes, pes cavus and severely reduced amplitudes of the compound motor nerve action potentials of the peroneal and tibial nerves, but preserved motor nerve conduction velocities. His mother, one brother and one sister reportedly have similar symptoms of peripheral neuropathy but denied further neurophysiological testing or genetic analysis.

The point mutation c.1040A>T in exon 11, leading to the exchange of Glutamine to Valine at position 347 (E347V) of the protein, was detected in a 32 year old male (fig. 1c, patient 3). The mutation was not observed by SSCP analysis in over 200 control chromosomes, detection by restriction analysis was not possible. The parents of the patient were reported to be healthy and unrelated, there is no history of peripheral neuropathies in the family. The patient has distal atrophy of his legs since childhood. He also shows high-grade paresis of peroneus muscles in both legs, severe pain in both legs and distal sensory loss. Electrophysiological studies indicate an axonal neuropathy with mildly decreased mNCV.

Patient 4, a 27 year old female, presented the mutation c.1403G>A in exon 14 (fig. 1d). The mutation leads to the exchange of Arginine to Histidine (R468H). Clinically the patient shows distal weakness and atrophy of the legs, distal sensory loss and decreased mNCV. Her father has Parkinson disease and distal neuropathy. Her paternal grandfather had pes cavus. Her sister and her mother both do not present any symptoms of neuropathy. Mutation analysis in the parents by restriction with *Aci*I and by sequencing proved the same mutation in her father, indicating cosegregation. However, the mutation was also detected in one of 260 control chromosomes.

A 44 year old female patient (patient 5) with symptoms of peripheral neuropathy since the age of six was found to carry a point mutation c.2113G>A in exon 18 (fig. 1e) leading to the exchange of Valine to Isoleucine at position 705 (V705I). No mutation was found in 212 control chromosomes by restriction digest with *Hin*I (Fermentas). Her deceased father and her brother were reported to have similar symptoms, but family members were not available for genetic analysis. Patient 5 shows distal weakness and atrophy of the lower and upper extremities. She has drop feet and steppage gait, absent Achilles' tendon reflexes, distal sensory loss of the lower extremities and decreased sensitivity to vibration of both ankles.

Patient 6 carries a frameshift mutation (c.2258_2259insT (L753fs)) in exon 19 (fig. 1f). This frameshift mutation causes a change of the last four amino acids of the open reading frame and leads to an extension of five new amino acids at the end of the protein. By restriction analysis of exon 19 with the enzyme *Pst*I, this mutation was not observed in 200 control chromosomes. Molecular genetic analysis was carried out for this patient at the age of 73 years. There is no family history of peripheral neuropathy but he was analyzed under suspect of CMT2 because of symmetric distal weakness and increased distal paresis of the legs.

## Discussion

We detected *MFN2 *mutations in 6 patients out of 73 patients with a clinical diagnosis of axonal CMT2, indicating a rate of 8% similar to the findings around 10% up to 20% observed by other groups. None of these mutations has been previously published, and (except the mutation c.1403G→ A in patient 4) none of these has been found in unrelated controls.

Our data confirm the findings of dominant mutations in the *MFN2 *gene to be associated with CMT2A [5]. Recently, mutations in *MFN2 *have also been linked to two rare forms of hereditary neuropathy, namely HMSN V (with pyramidal signs) [8] and HMSN VI (with optic atrophy) [9]. Our patients with mutations in *MFN2 *have axonal polyneuropathy without additional symptoms. Severity of symptoms is relatively mild and the rate of clinical progression slow.

Although there are several hints linking peripheral neuropathies to the mitochondrial network, the function of mitofusins in peripheral nerves and also the role of *MFN2 *mutations in the pathogenesis of peripheral neuropathy is only partly understood.

Mitochondria are active organelles forming a single dynamic network whose continuity is maintained by a balance of fission and fusion events [10]. In both yeast and drosophila, mitochondrial fusion is controlled by the nuclear-encoded mitochondrial transmembrane GTPase fuzzy onions (Fzo) [11-13]. In humans and mice MFN2 and MFN1, two human homologues of Fzo exist and both are essential for embryonic development and mitochondrial fusion [13]. *MFN2 *is the second HMSN disease gene directly involved in the maintenance of the mitochondrial network. In contrast to mitofusins, overexpression of ganglioside-induced differentiation associated protein 1 (GDAP1) is interfering with mitochondrial fusion and induces fragmentation of mitochondria, and recessive mutations in *GDAP1 *have been found in patients with CMT4A [14,15].

**Figure 2:**
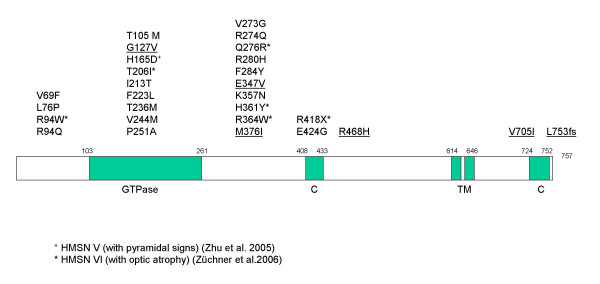
Structure of the MFN2 protein, showing functional domains and published mutations. Mutations described in this paper are underlined. Mutations associated with HMSN V and HMSN VI are marked with + and *, respectively.

MFN2 is localized to the outer mitochondrial membrane. The protein contains two coiled-coil regions flanking a transmembrane segment and a GTPase domain. Functional coiled-coil regions are essential for tethering of mitochondria before fusion [16-18]. An intact GTPase domain is indispensable for the function of mitofusins [12,17,19]. Overall, more than 25 different mutations in *MFN2 *have been observed up to now in patients with CMT2A, but also HMSN V and HMSN VI. There is currently no explanation for the divergence of phenotypes associated with mutations in *MFN2 *[5]. Many mutations are predicted to affect the GTPase domain of the protein, but an equal proportion has been detected for the region linking the GTPase domain and the first coiled coil region (see fig. 2). The mutations described up to now seem to cluster in several hotspot regions (aa94, aa236–251, aa273–284, aa357–364), but we have not found any recurrent mutation.

Five out of six mutations described in this paper are missense mutations, like the majority of mutations detected in *MFN2 *up to now [5-8]. Of the five missense mutations, one (G127V) affects the GTPase domain, and two mutations change the region linking the GTPase domain and the first coiled coil region (E347V and M376I). The two other missense mutations (R468H and V705I) are predicted to cause amino acid substitutions between the transmembrane domain and the coiled coil regions (fig. 2).

The missense mutation c.1403G→ A (R468H) is the first described in the region between the transmembrane domain and the C-terminal coiled coil region, and was detected in patient 4 and her symptomatic father, indicating cosegregation of mutation and disease. The same substitution was detected in one out of 130 anonymous control samples given by blood donors of different ages assumed to be healthy. Since mild symptoms of CMT2 may have been missed in a younger blood donor added to the control collective, this does not exclude the substition R468H as pathogenic, but it may also represent a rare sequence variant. Molecular biological studies may be necessary to finally determine the nature of this substitution.

Another novel mutation (c.1040A>T) found in patient 3 in our series causes the change of the polar glutamine to the non-polar valine at position 347. This amino acid exchange occurred in a highly conserved region of *MFN2*. This region is one of the indispensable segment of the protein that might provides binding sites for the assembly [18].

Only one stop mutation, R418X, has been described recently in a patient with CMT6 [9]. Interestingly, the frameshift mutation c.2258_2259insT in our patient 6 would result in a protein only differing by four amino acids and an extension of further five new amino acids at the C-terminus of the protein. This mutation may affect the C-terminal coiled-coil domain at the end of the fzo-mitofusin domain. Analysis of *MFN2 *deletion constructs revealed that GTPase-dependent interaction between the N-terminal and C-terminal tails of *MFN2 *through their coiled-coil domains and a highly conserved domain in the most N-terminal region is essential for mitochondrial fusion [18]. On the other hand it has been suggested that the C-terminus of MFN2 contains determinants required for targeting of the protein to the mitochondrial membrane [20].

Further studies of promoter domains in the *MFN2 *gene will help to clarify if these regions are involved in the development of CMT2.

## Conclusion

We confirmed a significant role of mutations in *MFN2 *in the pathogenesis of Charcot-Marie-Tooth disease type 2.

## Abbreviations

CMT: Charcot-Marie-Tooth

CMT2A: Charcot-Marie-Tooth type 2A

GDAP1: ganglioside-induced differentiation associated protein 1

KIF1B: kinesin family member protein 1B

MFN2: mitofusin 2

mNCV: motor nerve conduction velocity

SSCP: single strand conformation polymorphism

## Competing interests

The author(s) declare that they have no competing interests.

## Authors' contributions

KE carried out the molecular genetic studies, KE and MM participated in the sequence alignment and drafted the manuscript. MH participated and assisted in molecular genetic studies and the sequence alignment. MM conceived of the study, and participated in the design. JTE participated in its design and coordination and helped to draft the manuscript. MV, GH and JG made major contributions to the clinical characterisation of the patients. All authors read and approved the final manuscript.

## Pre-publication history

The pre-publication history for this paper can be accessed here:


